# 塞利尼索联合去甲基化药物治疗维奈克拉暴露难治/复发急性髓系白血病疗效分析

**DOI:** 10.3760/cma.j.issn.0253-2727.2023.11.009

**Published:** 2023-11

**Authors:** 剑 张, 宝全 宋, 欣 孔, 吟 刘, 翰麟 杨, 李红 宗, 金玉 孔, 洋 徐, 惠英 仇, 德沛 吴

**Affiliations:** 苏州大学附属第一医院血液内科，江苏省血液研究所，国家血液系统疾病临床医学研究中心，苏州 215006 The First Affiliated Hospital of Soochow University, Jiangsu Institute of Hematology, National Clinical Research Center for Hematologic Diseases, Suzhou 215006, China

维奈克拉联合去甲基化药物（HMA）方案在难治/复发急性髓系白血病（R/R AML）患者中的缓解率可达50％，然而仍有约1/3的患者对该方案原发耐药。维奈克拉联合HMA一线治疗失败的患者经挽救治疗后预后仍较差，中位生存期仅2.9个月[1]。维奈克拉耐药的机制复杂，另外R/R AML患者往往存在骨髓抑制、伴发感染等合并症，后续低强度治疗方案选择较为困难。塞利尼索（Selinxor）是第一个口服选择性核输出蛋白（XPO1）抑制剂[2]。既往研究表明，XPO1在AML中高表达，参与肿瘤的发生发展。塞利尼索竞争性抑制XPO1核输出功能，激活肿瘤抑制蛋白如p53、p21的功能；并降低细胞质中致癌蛋白的水平，如C-kit、BCL2和MCL1，发挥抗白血病活性[3]。本回顾性研究中我们分析了12例接受塞利尼索联合HMA地西他滨（DAC）、阿扎胞苷（AZA）治疗的R/R AML患者的临床资料，初步观察探讨其疗效和安全性。

## 病例与方法

1. 病例：纳入2021年5月1日至2022年12月31日在苏州大学附属第一医院血液科接受治疗的12例既往维奈克拉治疗过的R/R AML患者。12例患者均于初诊时行MICM分型（骨髓细胞形态学、流式细胞术检测白血病免疫表型、细胞遗传学、融合基因和基因突变组套等检查）。参照NCCN指南标准[4]、2022ELN修正AML[5]诊断分层。

2. 治疗方案：塞利尼索+HMA方案具体为：塞利尼索 40 mg，每周2次，口服，连用3周，停1周。28 d为1个疗程。DAC 10 mg/d，7～10 d，静脉输注或 AZA 75 mg·m^−2^·d^−1^，第1～7天，皮下注射（大于60岁患者剂量减半）。其中2例难治患者流式细胞术检测CD38表达大于50％，加用达雷妥尤单抗16 mg/kg每周1次×2周。治疗期间，依据患者临床指征给予抗感染、输注成分血、重组人G-CSF、止吐等支持治疗以及进行相应剂量调整。部分患者行异基因造血干细胞移植。

3. 疗效评估：早期死亡定义为诱导治疗开始30 d内任何原因造成的死亡。所有患者化疗结束21～28 d复查骨髓，结合血常规结果判断疗效。依据2021年我国成人急性髓系白血病诊疗指南[6]及2003年国际工作组的推荐[7]，进行疗效评估。总体反应率（ORR）为完全缓解（CR）率、伴不完全血液学恢复的完全缓解（CRi）率、血小板未恢复的完全缓解（CRp）率、骨髓无白血病状态（MLSF）率、部分缓解（PR）率之和，复合CR（CRc）率为CR率、CRi率、CRp率之和。复发和难治的定义参见文献[8]。

4. 随访：采用电话、查阅门诊/住院病历进行患者生存情况随访。随访截至2022年12月31日，中位随访时间8.2（1.5～16.0）个月。无病生存（DFS）期定义为从塞利尼索联合HMA方案治疗后达CR之日至复发、死亡、接受造血干细胞移植或随访截止日为止。总生存（OS）期定义为从塞利尼索联合HMA方案开始至死亡或者随访终止时间。

5. 统计学处理：统计学分析使用SPSS 26.0软件，采用Kaplan-Meier法进行生存分析。

## 结果

1. 临床特征：12例既往接受过维奈克拉治疗的R/R AML患者的临床基本特征及合并症见表1。12例患者中男7例，女5例。中位年龄36（18～68）岁。年龄大于65岁患者2例。7例为难治性AML，5例为复发AML（包括1例移植后复发）。FAB分型：M_1_ 1例，M_2_ 9例，M_4EO_ 1例，M_5_ 1例。ELN危险分层：预后中等3例，预后不良9例。染色体：正常核型4例，复杂/单体核型6例，其他核型2例。合并症：既往血液病史3例，感染8例，脏器功能不全2例。联合DAC 8例，联合AZA 4例。既往应用HMA患者7例，未应用5例。所有患者既往接受至少1个疗程常规化疗或初诊评估不适合强化疗患者予维奈克拉联合HMA方案诱导治疗。

2. 塞利尼索+HMA方案疗效：所有患者均可评估疗效。12例R/R AML患者ORR为66.7％（8/12），CRc率为50％（6/12），6例达CR或CRi患者中3例微小残留病（MRD）≤1×10^−3^。7例难治性患者6例获得治疗反应，5例复发患者2例获得治疗反应。3例初诊预后低中危患者2例获得治疗反应，9例预后高危患者4例获得治疗反应。2例加用达雷妥尤单抗患者中1例1个疗程后获得MRD（+）CRi；另1例疗效评估为MLFS。7例（58.3％）患者桥接异基因造血干细胞移植，移植患者中6例截至随访疾病处于MRD（−）CR状态存活，1例移植后合并重症肺部感染死亡。4例塞利尼索联合AZA组患者2例获得治疗反应，8例塞利尼索联合DAC患者6例获得治疗反应。患者治疗方案、疗程及治疗反应见表1。

**表1:** 12例塞利尼索联合甲基化药物（HMA）方案治疗难治/复发AML患者的临床特征、疗效评估

例号	年龄（岁）	性别	诊断	2022ELN预后危险度分层	染色体核型	基因突变	复发性或难治性	骨髓原始细胞^a^（%）	MRD^a^（%）
1	68	女	AML	预后不良	复杂核型/单倍体核型	NRAS, NF1, BCORL1, CARD11, CBFB-MYH11	难治	42.0	27.2
2	68	女	AML(MDS转化）复发	预后不良	复杂核型/单倍体核型	IDH1, DNMT3A	复发	20.0	34.3
3	52	男	AML难治	预后不良	其他核型	MLL-AF6, KRAS, NRAS	难治	38.0	3.9
4	45	男	AML移植后复发	预后不良	正常核型	MLL-AF9, WT1, EVI1, NRAS, PTPN11, BRCA1	复发	79.0	50.2
5	35	男	AML 复发	预后中等	正常核型	CEBPA, FLT3-ITD	复发	10.5	22.4
6	33	男	AML-MF	预后不良	复杂核型/单倍体核型	TP53, ZAP70	难治	11.0	12.0
7	37	男	AML复发	预后中等	其他核型	RUNX1-RUNX1T1, WT1, EVI1, KIT, FLT3-ITD	复发	32.5	30.0
8	28	男	AML	预后不良	正常核型	NF1 Q209fs, SF3B1 K700E	难治	10.0	6.6
9	18	男	AML	预后不良	复杂核型/单倍体核型	TLS-ERG, WT1, TET2, PAX5, ZMYM3	难治	55.5	24.4
10	35	女	AML复发	预后不良	复杂核型/单倍体核型	KMT2A-SEPT6, PTPN11, CSMD1	复发	84.0	84.8
11	25	女	AML(MDS转化）	预后中等	正常核型	NF1	难治	69.0	23.8
12	54	女	AML(MDS转化）	预后不良	复杂核型/单倍体核型	CEBPA, RUNX1, SRSF2, TET2	难治	10.0	10.5

例号	塞利尼索（X）联合HMA方案	疗效评估	移植类型	转归	随访时间（月）
1	X 40 mg biw×3周 AZA 75 mg/m^2^ qd×7 d，2个 疗程	MRD（+）CR	未移植	死亡	16
2	X 20 mg biw×3周 DAC 10 mg qd×7 d，1 个疗程	NR	未移植	死亡	15
3	X 40 mg biw×3周 AZA 75 mg/m^2^ qd×7 d，1个疗程	MRD（−）CRp	Haplo-HSCT	死亡	5
4	X 40 mg biw×3周 AZA 75 mg/m^2^ qd×7 d，1个 疗程	NR	Haplo-HSCT	死亡	9
5	X 40 mg biw×3周 DAC 10 mg qd×10 d，1个疗程	MRD（−）CR	Haplo-HSCT	存活	10
6	X 40 mg biw×3周 DAC 10 mg qd×10 d，1个疗程	MRD（+）CRi	Haplo-HSCT	存活	9
7	X 40 mg biw ×3周 DAC 10 mg qd×10 d，1个疗程	MRD（−）CR	Haplo-HSCT	存活	7
8	X 40 mg biw×3周 AZA 75 mg/m^2^ qd×7 d，1个疗程	NR	未移植	死亡	6
9	X 40 mg biw×3周 DAC 10 mg qd×10 d，1个疗程 daratumumab 16 mg/kg qw×2周	MRD（+）CRi	Haplo-HSCT	存活	5
10	X 40 mg biw×3周 DAC 10 mg qd×10 d，1个疗程	NR	未移植	死亡	1.5
11	X 40 mg biw×3周 DAC 20 mg/m^2^ qd×5 d,1个疗程 daratumumab 16 mg/kg qw×2周	MLFS	Haplo-HSCT	存活	2.5
12	X 40 mg biw×5周 DAC 10 mg qd×10 d，1个疗程	MLFS	MSDT	存活	2.5

注 ^a^塞利尼索联合HMA方案治疗前；MRD：微小残留病；AML：急性髓系白血病；MDS：骨髓增生异常综合征；AZA：阿扎胞苷；DAC：地西他滨；daratumumab：达雷妥尤单抗；biw：每周2次；qd：每日1次；qw：每周1次；CR：完全缓解；CRi：伴不完全血液学恢复的完全缓解；CRp：血小板未恢复的完全缓解；MLSF：骨髓无白血病状态；NR：未缓解；Haplo-HSCT：单倍型造血干细胞移植；MSDT：HLA相合同胞供者移植

3. 生存情况：12例R/R AML患者中位随访时间为8.2个月。6个月OS率为62.9％，中位OS期5个月，中位DFS期4.5个月。截至随访时间，6例存活，6例死亡。

4. 不良反应：12例患者治疗后均出现骨髓抑制。10例患者出现3或4级中性粒细胞减少，7例出现3或4级血小板减少。中性粒细胞缺乏恢复（≥0.5×10^9^/L）的中位时间为14（8～21）d。血小板减少恢复（≥20×10^9^/L）的中位时间为16（8～24）d。8例患者治疗过程中出现粒细胞缺乏（粒缺）伴感染症状：1例移植后复发患者合并重症肺部感染，3例伴上呼吸道感染，2例伴肛周感染，2例伴粒缺期发热。除1例移植后重症肺部感染病例死亡外，其余患者经全身及局部抗感染治疗好转，使用抗生素的中位时间为14（9～35）d。8例（66.7％）患者出现胃肠道反应，无急性移植物抗宿主病（aGVHD）发生，肝肾功能未见异常。

## 讨论

初治不适合强化疗患者治疗选择有限，维奈克拉联合方案可显著改善临床反应、延长生存期，成为其标准一线治疗方案[9]–[10]。然而维奈克拉治疗失败或者复发的患者，预后非常差，中位OS期不足3个月[11]。研究表明对维奈克拉联合用药的原发性耐药或适应性耐药与多种复杂因素相关，包括FLT3-ITD、RAS/MAPK突变和TP53突变扩增、烟酰胺代谢升高、c-myc、MCL1代偿性高表达等[12]。对于这类患者的挽救治疗，迫切需要由无交叉耐药的新药组成新联合疗法。

塞利尼索是口服的XPO1抑制剂，AML细胞中存在XPO1突变或过表达，且与生存期缩短显著相关。抑制XPO1能够阻止真核起始因子4E（eIF4E）介导的BCL2和MCL1 mRNA出核翻译，从而同时下调BCL2和MCL1蛋白表达，诱导AML细胞凋亡，与维奈克拉联合可协同增强抗白血病活性[13]。基于上述基础研究，塞利尼索一定程度上能够克服维奈克拉的相关耐药。

对于维奈克拉难治的、无法耐受化疗的R/R AML人群，仍有待探索塞利尼索相关联合方案。DAC可通过上调CDKN1A和FOXO3A表达，增强塞利尼索的抗白血病活性[14]；而塞利尼索与AZA联合可促进P53核聚积诱导骨髓增生异常综合征（MDS）细胞株凋亡[15]。本研究中采用的是塞利尼索同时联合HMA的给药方式，针对经维奈克拉联合方案治疗后复发或进展的R/R AML患者，整体CRc率50％，ORR 66.7％，中位OS期为5个月，中位DFS期为4.5个月。疗效均有一定改善且化疗后骨髓抑制期并未见明显延长。此方案后续有58.3％（7/12）的患者桥接异基因造血干细胞移植，移植后患者疾病达到MRD阴性的CR状态，为患者争取更好的长期生存机会，且并未增加aGVHD发生率。提示该挽救治疗方案后续桥接异基因造血干细胞移植可作为复发难治的不适合强化疗AML患者的治疗选择。

本研究纳入了既往接受过维奈克拉治疗后进展的R/R AML患者，其中58％（7/12）患者曾接受过HMA治疗，在接受塞利尼索联合HMA方案治疗后CRc率仍有42.9％（3/7），而既往未接受过HMA的患者CRc率更达100％。提示既往未经HMA治疗的患者从塞利尼索联合HMA治疗中获益更多。

伴有TP53等基因突变、−7以及复杂染色体核型的AML患者预后往往较差[16]。虽然本研究中因纳入患者较少，无法就特定的突变基因的反应总结出明确结论，但6例存在复杂染色体核型的患者中3例可获得CR，显示出较好的疗效。另外1例TP53突变、2例FLT3-ITD低频突变，以及存在融合基因（MLL-AF6、TLS-ERG、RUNX1-RUNX1T1）的这类预后较差的患者仍可通过本方案获得CR。2例继发性AML（MDS转化）及1例合并骨髓纤维化患者疗效评估获得CRi/MLSF缓解状态。以上值得进一步关注。

R/R AML患者应用塞利尼索联合HMA方案疗效较好，不良反应可耐受。其中伴有高危基因突变及复杂染色体核型的患者，短期疗效尚可，桥接异基因造血干细胞移植可使患者获得长期生存机会，值得长期持续关注。本研究是单中心、小样本量的初步回顾性分析，还需要扩大样本量、延长随访时间进行验证和分析。
